# Effect of intra-nasal nitrilotriacetic acid trisodium salt in lowering elevated calcium cations and improving olfactory dysfunction in COVID-19 patients

**DOI:** 10.1007/s00405-022-07424-5

**Published:** 2022-05-14

**Authors:** Mohamed H. Abdelazim, Ahmed H. Abdelazim, Wael F. Ismaiel, Mahmoud E. Alsobky, Ahmed Younes, Abdelgawad M. Hadeya, Sherif Ramzy, Mohammed Shahin

**Affiliations:** 1 Department of Otolaryngology, Faculty of Medicine, Al-Azhar University, Damietta, 34518 Egypt; 2 Pharmaceutical Analytical Chemistry Department, Faculty of Pharmacy, Al-Azhar University, Nasr City, Cairo, 11751 Egypt; 3 Pharmaceutical Analytical Chemistry Department, Faculty of Pharmacy, Damanhour University, Beheira, Egypt

**Keywords:** Smell, Anosmia, COVID-19, SARS-CoV-2

## Abstract

**Purpose:**

An association between COVID-19 and olfactory dysfunction has been noted in many patients worldwide. The olfactory adaptation process leads to an increase in intracellular calcium cation levels. Nitrilotriacetic acid trisodium salt has high selective chelation for calcium cations from olfactory mucus. The aim of this work is to test the effect of an intranasal nitrilotriacetic acid trisodium salt to lower the elevated calcium cations in COVID-19 patients with relevant symptoms of olfactory dysfunction.

**Methods:**

Fifty-eight COVID-19 adult patients with relevant symptoms of olfactory dysfunction were enrolled in a prospective randomized controlled trial. They received a nasal spray containing either 0.9% sodium chloride or 2% nitrilotriacetic acid trisodium salt. Olfactory function was assessed before and after treatment using the Sniffin’ Sticks test. Quantitative analysis of calcium cation concentration in nasal secretions was performed using a carbon paste ion-selective electrode.

**Results:**

After the application of nitrilotriacetic acid trisodium salt compared to sodium chloride, a significant improvement from functional anosmia to healthy normosmia with significant decrease in calcium cation concentration was observed.

**Conclusions:**

Further collaborative research is needed to fully investigate the effect of an intranasal nitrilotriacetic acid trisodium salt in the treatment of olfactory disorders.

## Introduction

Post viral anosmia has been frequently noted after a variety of upper respiratory tract infections. Identification of the exact pathogenesis remains unclear. Recently, an association between COVID-19 and anosmia has been reported worldwide.

There are several observations showing that anosmia may be the only sign of COVID-19 without the presence of other clinical symptoms. Since the long-term effects of anosmia in COVID-19 have been studied, there was an urgent need to investigate treatment alternatives for anosmia related COVID-19 infection [1–3].

In the response to odorants in intact neurons, reports demonstrated the essential role of calcium in the olfaction transduction process, including inhibitory feedback. The inhibitory role of calcium was thought to be due to two mechanisms. First, calcium–calmodulin interacted with the channels controlled by cyclic nucleotides, leading to lower sensitivity of these channel to cyclic nucleotides and thus decreased positive current influx. The second mechanism was thought to be related to calcium-dependent phosphorylation and consequent inhibition of adenylyl cyclase, leading to a decrease of intracellular cyclic adenosine monophosphate and decreased activation of cyclic nucleotide-gated channels. The latter of these two mechanisms could be important for the adaptation of the olfactory response to prolonged stimulus exposure. More specifically, an increase in the mucosal calcium levels could cause desensitization of the olfactory receptor neurons. Therefore, it is hypothesized that the changes in odor sensitivity caused by small changes in mucosal calcium levels can significantly alter the sensitivity of cyclic nucleotide-gated channels and thus the excitability of receptor neurons in vivo, leading to an improvement in the sense of smell [4–6]. Nitrilotriacetic acid trisodium salt (NTA), a calcium chelating agent, has the ability to bind free calcium ions in the nasal mucus and could somehow modulate these inhibitory effects, thereby improving the olfaction process. In general, NTA is a triple tetradentated trianionic chelating agent that forms a stable complex with various metals. It can chelate metal ions that often cause water hardness, such as calcium and magnesium cations [7]. It is used in cleaning products, industrial water treatment and textile treatment. Also, it has specific use in the formulation of cosmetic products to remove calcium and magnesium cations that affect foaming and cleaning performance and cause turbidity in clear liquids [8]. NTA has a high selective sequestration capacity for calcium cations and forms a stable complex product. This suggests that NTA can reduce intranasal free calcium cation and subsequently improve olfactory function in patients with anosmia COVID-19 infection.

In this work, we tested the effect of intranasal NTA on reducing the increase of calcium cations in the mucus of patients with anosmia associated with COVID-19. This is the first published clinical trial testing NTA for the treatment of anosmia in COVID-19 patients.

## Methods

### Study design

Prospective randomized blinded double controlled clinical trial was conducted at the ENT Department of Damietta Faculty of Medicine, Al Azhar University, Egypt. The study was approved by the Ethical Committee of Damietta Faculty of Medicine, Al-Azhar University (IRB00012367-20-06-011). Patients were randomly divided into two groups, sodium chloride and NTA, by unratified block randomization with a block size of four. A computerized randomization plan was developed to ensure block randomization. The allocation and organization of the groups were blinded to the physicians and the patients. The protocol book remained in the hands of a designated team member who did not communicate with the patients or the interviewer.

### Patient selection

Fifty-eight COVID-19 adult patients with relevant anosmia symptoms, 30 females and 28 males, were enrolled in the study during a period from June 2020 to September 2020. Written informed consent was obtained from participants of the proposed study. Only COVID-19 participants over 18 years of age with more than 14 days of olfactory dysfunction were included. Descriptive characteristics of the study participants were recorded.

### Treatment regimen

Patients were divided into two equal groups: intranasal 0.9% sodium chloride (group A) and intranasal 2% NTA in phosphate buffer, pH 6 (group B). Intranasal pharmaceutical preparations of the described treatment were prepared by the Department of Pharmaceutical Analytical Chemistry, Cairo Faculty of Pharmacy, Al-Azhar University. Regarding to previously report for NTA human metabolism and its importance in the total safety evaluation program, no adverse health effects were reported in a metabolism study in which volunteers ingested a single dose of 10 mg of NTA [8]. In the described study, NTA was prepared as 2% in phosphate buffer solution to produce final definite solution labeled to contain 20 mg/mL NTA. The formulations were provided to deliver a standardized volume of 0.1 mL (2 mg/mL NTA) for every dose, so.it was considered to be safe administered nasal formulation. These formulations were filled into opaque nasal spray bottles to deliver 0.1 mL of the formulated spray solutions. The bottles were labeled with a specific sealed code that was not accessible to team members involved in the study. They remained sealed unless there was a need to detect secrecy due to adverse effects presented by a participant. The treatment was administered to the participants three times daily for 1 month. The sodium chloride group, the control group, was used to exclude any difference between the treated and non-treated groups.

### Olfactory function assessment

Olfactory function check was performed with “Sniffin’ Sticks” test (Burghart Messtechnik, Wedel, Germany). Four values, threshold (T), discrimination (D), identification (I) and the TDI value were measured before treatment and 1 month later [9]. A TDI score below 16.75 points represents functional anosmia. Also, a TDI score between 16.75 and 30.50 points represents hyposmia, and a TDI score of 30.75 points or more represents normosmia. The patient is considered improved when the TDI score has increased by 6 points [10].

### Quantitative determination of calcium cations of the nasal secretions

Participants’ nasal secretions were collected before treatment and 1 month later immediately after sneezing. For this purpose, a small stainless steel clamp (approximately 10 mm × 5 mm × 2 mm) was clamped on the septum between the nostrils so that the secretions could drain into a special 1.5-mL tube [11]. The collected nasal secretions were transferred to settled centrifugation tubes after addition of 0.5 mL phosphate buffer solution. The protein layer was completely denatured by the addition of 3 mL acetonitrile, centrifuged at 4000 rpm for 40 min, and finally evaporated to dryness. Then the residue layer was diluted with phosphate buffer solution in 10-mL specific tubes. The carbon paste ion-selective electrode [12] was developed by the Department of Pharmaceutical Analytical Chemistry, Cairo Faculty of Pharmacy, Al-Azhar University. It was used to quantitatively analyze the calcium cation concentration of the collected nasal fluid samples from all participants before treatment and 1 month later.

### Statistical analysis

Statistical tests were performed using SPSS v23 statistical software (SPSS, Inc, Chicago, Illinois). Results obtained were expressed as mean ± standard deviation unless otherwise stated. Differences in frequencies were tested using Fisher’s exact probability test. Paired and unpaired Student’s *t* tests were calculated to test the significance of the results obtained. Statistical significance was assigned when *p* < 0.05.

## Results

Fifty-eight COVID-19 patients with relevant olfactory dysfunction were included in this prospective study. The age of the patients ranged from 18 to 55 years. There were 30 females and 28 males. The complete characteristics of the patients are described in Table 1. Fisher’s exact probability test was used to assess the sample size. The results were not significant between the sodium chloride group and the NTA group, as shown in Table 1.

**Table 1:** Patient’s characteristics

Character	Sodium chloride	NTA	*p* (Fisher exact probability test)
Sample size, *n*	29	29	
Age (years), mean ± SD	39.87 ± 6.58	38.67 ± 7.21	
Days since symptoms to enrollment, mean ± SD	16.24 ± 1.15	16.45 ± 1.28	
Gender (male/female), *n*	14/15	16/13	
Smokers (current/never), *n*	5/29	4/29	1.00
Comorbidities, *n*			
Asthma	2	3	1.00
Diabetes	6	7	1.00
Hypertension	5	6	1.00
Migraine	4	2	0.67
Sinusitis	2	3	1.00

**Fig. 1:**
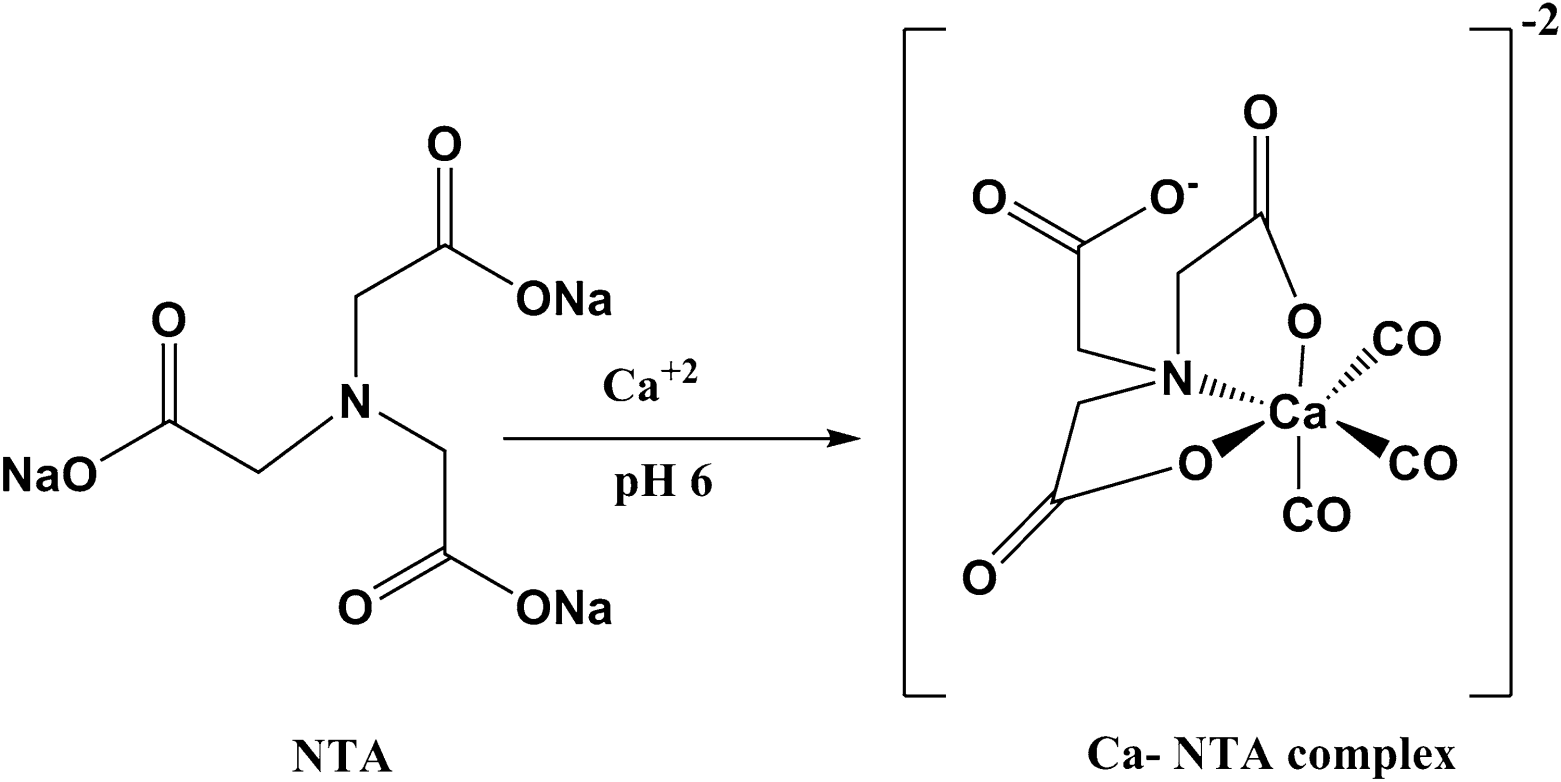
Shematic reaction pathway of nitrilotriacetic acid trisodium and calcium cation

Intra-nasal administration of NTA had the ability to form a calcium–NTA complex and decreased the concentrations of calcium cations in the olfactory mucus of the participants. NTA had the selective ability to chelate calcium cations at a specific pH of 6 and form stable complex products. The mechanism for the reaction of NTA with calcium cations is shown in Fig. 1.

Olfaction was evaluated using the Sniffin’ Sticks test before and after treatment with sodium chloride or NTA. The values of T, D, I, and TDI were measured before treatment and 1 month later. Mean values were also calculated and presented in Table 2. Comparison of TDI values with reference values was performed. The results showed that the mean ± SD of TDI value after treatment with sodium chloride was 19.66 ± 1.67, Table 2. Also, the mean ± SD of TDI value after treatment with NTA was 31.91 ± 1.56, Table 2. The results showed improvement in patients receiving sodium chloride from functional anosmia to hyposmia and great improvement in patients receiving NTA from functional anosmia to healthy normosmia.

**Table 2:** Results and statistical assessment before and after intranasal application of sodium chloride and NTA

	Sodium chloride		Nitrilotriacetic acid trisodium	
	Pre administration	Post administration	Pre administration	Post administration
Threshold (T) score, mean ± SD	2.49 ± 0.48	3.40 ± 0.87	2.55 ± 0.41	9.82 ± 0.953
Discrimination (D) score, mean ± SD	4.05 ± 1.01	8.13 ± 0.98	4.11 ± 0.72	10.70 ± 0.84
Identification (I) score, mean ± SD	3.79 ± 0.79	8.13 ± 1.10	4.92 ± 0.98	11.39 ± 1.02
TDI score, mean ± SD	10.33 ± 1.40	19.66 ± 1.67	11.58 ± 1.21	31.91 ± 1.56
Calcium cation concentration (mM), mean ± SD	37.08 ± 2.12	23.04 ± 2.07	37.25 ± 2.17	11.50 ± 2.63

The concentration of calcium cations was quantitatively analyzed in the olfactory mucus of the patient. A carbon paste electrode was developed for potentiometric analysis of calcium cations. Electromotive force values were measured over a calcium concentration range of 100–0.001 mM to obtain a calibration plot relating electromotive force values to the negative log of calcium cation concentration. The designed electrode showed a Nernst slope of 28.73 mV/decade with a detection limit of 0.0001 mM in a linear range from 100 to 0.001 mM calcium cation concentration. The concentrations of calcium cations in the collected nasal secretions were successfully determined using the developed electrode. The results showed that the mean ± SD of calcium cation concentration (mM) after sodium chloride was 23.04 ± 2.07, Table 2. Also the mean ± SD of calcium cation concentration (mM) after NTA was 11.50 ± 2.63, Table 2. The results showed a significant decrease in calcium cation concentration in the patients treated with NTA.

Statistical analysis with the paired *t* test was used to compare the means and standard deviations of TDI values for the NTA group before and after treatment. The results showed that NTA resulted in significant improvement in the olfactory performance, *t* (23) = 46.90, *p* = 3.5 × 10^–9^. The result was significant at *p* < 0 0.05. In addition, an unpaired *t*-independent test was used to compare the mean TDI values of the NTA group with the sodium chloride control group. The result was significant, *t* (46) = 25.93, *p* = 1.8 × 10^–17^.

## Discussion

A disturbance of the sense of smell is one of the most common symptoms associated with many cases of corona virus. Several reports indicate that elevated calcium cation levels in olfactory mucus have adverse effects on the olfactory mechanism. The shift in calcium cation concentration has physiological significance and correlates with the odor management process [13, 14].

During the response to odorants, there is a simultaneous increase in cyclic adenosine monophosphate and calcium cations. The increase in cyclic adenosine monophosphate is due to activation of adenylate cyclase. Similarly, the increase in intracellular calcium cations is due to influx into the cyclic nucleotide channels. Therefore, it is hypothesized that a decrease in intranasal free calcium cations may reduce feedback inhibition and lead to improved olfactory function [4–6].

This prospective randomized controlled trial tested the intranasal use of NTA for the treatment of olfactory dysfunction in COVID-19 patients. NTA is a chelating agent with three possible coordinating sites, two carboxylates and one amine group, enabled the complexation with a metal ion. This occurred when calcium cations and NTA salts were combined in aqueous solutions [7, 8]. At a pH of 6, NTA selectively forms a calcium-NTA complex even in the presence of sodium, potassium or magnesium cations. This chemical process leads to a reduction of calcium cations in olfactory mucus patients. The proposed results suggest that the reduction of calcium cations in olfactory mucus by NTA improves olfactory function.

The Sniffin Sticks test is a widely used tool for evaluating olfactory function that consists of three subtests: odor threshold, odor discrimination, and odor identification. The Sniffin Sticks test was introduced over two decades ago and has proven to be highly reliable and valid. The calculated results of the odor test confirmed that the application of NTA produces a significant improvement in the olfactory performance.

Electrochemical determination of calcium cations in olfactory mucus provides evidence of an increase or decrease in calcium cations. The carbon paste electrodes are among the ion-selective electrodes with the unique advantages of robustness, stable response and suitability for a wide range of detection applications. Moreover, they can be used with a small volume of nasal secretion [15]. The results obtained showed that the calcium cation concentration decreased sharply in the participants treated with NTA. This could be explained by the chelation of the calcium cation and the formation of the calcium–NTA complex product. The proposed work provided evidence for the relationship between the reduction of calcium cation concentration in olfactory mucus by NTA and the improvement of olfactory performance.

The main limitation of this study is the small sample size, which predisposed to an underpowered analysis. Actually, it will be important to extend this study for more studied populations and expand this study for olfactory dysfunction related to various factors rather than COVID-19 infection.

## Conclusions

In this work, the effect of intranasal nitrilotriacetic acid trisodium salt on reducing an elevated calcium cation in the mucus of COVID-19 with olfactory dysfunction was tested. After the application of nitrilotriacetic acid trisodium salt compared to sodium chloride, a significant improvement from functional anosmia to healthy normosmia with significant decrease in calcium cation concentration was observed. Based on the study described, intranasal administration of nitrilotriacetic acid trisodium salt may be a useful specific treatment for olfactory dysfunction associated with COVID-19.

## Declarations

### Trial registration

Registered with the Ethical Committee of Damietta Faculty of Medicine, Al-Azhar University under trial number IRB00012367-20-06-011.
